# Supplementary material for: Optomechanically induced transparency of x-rays via optical control
Source: Sci Rep. 2017 Mar 23;7:321. doi: 10.1038/s41598-017-00428-w (PMC5428473; doi:10.1038/s41598-017-00428-w)
Supplement: Supplementary file 1 — Supplementary information [file 41598_2017_428_MOESM1_ESM.pdf]

Optomechanically induced transparency of x-rays via optical control: Supplementary Information

Wen-Te Liao[1,2] and Adriana Pálffy[1]

[1]Max-Planck-Institut für Kernphysik, Saupfercheckweg 1, 69117 Heidelberg, Germany

[2]Department of Physics, National Central University, 32001 Taoyuan City, Taiwan

In the following we present in detail the analytic derivations.

TABLE S1: Symbol table.

Symbol	Explanation
$\hat{a}$	cavity optical photon annihilation operator
$\hat{a}^\dagger$	cavity optical photon creation operator
$\hat{b}$	phonon annihilation operator for the movable microlever
$\hat{b}^\dagger$	phonon creation operator for the movable microlever
$\hat{x}$	x-ray photon annihilation operator
$\hat{x}^\dagger$	x-ray photon creation operator
ω_c	resonant angular frequency of the optical cavity
ω_n	nuclear transition angular frequency
Ω	Rabi frequency coupling between the nuclear transition currents and the x-ray
k_x	x-ray wave vector
ω_x	x-ray photon angular frequency
ω_l	optical laser angular frequency
Δ_c	$=\omega_l-\omega_c$ optical laser detuning to the cavity angular frequency
Δ	$=\omega_x-\omega_n$ the x-ray detuning to the nuclear transition angular frequency
P	optical laser power
$\hbar$	reduced Planck constant
m	mass of the movable microlever
L	cavity length
δL	$=\hbar\omega_c\bar{n}_{cav}/(Lm\omega_0^2)$ averaged cavity length shift

ω_0	inherent oscillation (phonon) angular frequency of the movable microlever
$\delta\omega_0$	$= 4G^2\left(\frac{\omega_0}{\kappa^2+16\omega_0^2}\right)$ microlever's optomechanically induced oscillation angular frequency shift
ω_m	$= \omega_0 + \delta\omega_0$ optomechanically modified oscillation angular frequency of the microlever
Γ	spontaneous decay rate of nuclear excited state
κ	optical cavity photon decay rate
γ_0	inherent mechanical damping rate of the microlever
$\delta\gamma_0$	$= 4G^2\left(\frac{1}{\kappa} - \frac{\kappa}{\kappa^2+16\omega_0^2}\right)$ optomechanically induced mechanical damping rate shift
γ_m	$= \gamma_0 + \delta\gamma_0$ optomechanically modified mechanical damping rate of the microlever
s	$= \frac{\Gamma}{2} + \kappa + \gamma_m$ total decoherence rate of the system
$\overline{n}_{cav}$	$= \frac{\kappa P}{\hbar\omega_l\left[(\omega_l-\omega_c)^2+(\kappa/2)^2\right]}$ averaged cavity optical photon number
Y_{ZPF}	$= \sqrt{\hbar/(2M\omega_m)}$ zero-point fluctuation of the movable microlever
G_0	$= \omega_c Y_{\mathrm{ZPF}}/L$ optomechanical coupling constant
G	$= G_0\sqrt{\overline{n}_{\mathrm{cav}}}$ optomechanical coupling constant in the perturbation region
η	$= k_x Y_{\mathrm{ZPF}}$ Lamb-Dicke parameter
F_n^m	$= \langle m\|e^{i\eta\left(\widehat{b}^\dagger+\widehat{b}\right)}\|n\rangle$ Franck-Condon coefficient

HAMILTONIAN

The full Hamiltonian of the system sketched in Fig. 1a is a combination of the optomechanical Hamiltonian $\widehat{H}_{\mathrm{opto}}$ and nuclear interaction with x-ray photons $\widehat{H}_{\mathrm{nx}}$

$$\widehat{H} = \widehat{H}_{\mathrm{opto}} + \widehat{H}_{\mathrm{nx}}. \tag{S1}$$

Here

$$\widehat{H}_{\mathrm{opto}} = \hbar\omega_0\widehat{b}^\dagger\widehat{b} + \hbar\omega_c\widehat{a}^\dagger\widehat{a} - \hbar G_0\widehat{a}^\dagger\widehat{a}(\widehat{b}^\dagger + \widehat{b}), \tag{S2}$$

$$\widehat{H}_{\mathrm{nx}} = \hbar\omega_n|e\rangle\langle e| + \frac{\hbar\Omega}{2}\left(e^{-i\omega_x t + ik_x Y_{\mathrm{ZPF}}\left(\widehat{b}^\dagger+\widehat{b}\right)}\widehat{x}|e\rangle\langle g| + e^{i\omega_x t - ik_x Y_{\mathrm{ZPF}}\left(\widehat{b}^\dagger+\widehat{b}\right)}\widehat{x}^\dagger|g\rangle\langle e|\right), \tag{S3}$$

which are standard expressions and can be found in Refs. [1] and [33,36], respectively.

Derivation of Equation (3)

In order to derive the expression of the Hamiltonian in Eq. (S1) in the interaction picture, an unitary transformation to the rotating frame [1] can be done by using $\widehat{H}_{\mathrm{in}} = \widehat{U}\widehat{H}_{\mathrm{old}}\widehat{U}^\dagger - i\hbar\widehat{U}\frac{\partial\widehat{U}^\dagger}{\partial t}$ and

$\widehat{U} = exp\left(i\omega_l \widehat{b}^\dagger \widehat{b} t + i\omega_x \widehat{x}^\dagger \widehat{x} t\right)$. Moreover, as the quantum state of x-rays used in nuclear scattering experiments can be approximated by a coherent state, we invoke the semiclassical treatment for coupling between the x-ray and nuclei, namely, $\Omega\widehat{x} \rightarrow \Omega$. Both derivations give

$$\widehat{H} \;=\; \hbar\omega_0 \widehat{b}^\dagger \widehat{b} - \hbar\Delta_c \widehat{a}^\dagger \widehat{a} - \hbar G_0 \widehat{a}^\dagger \widehat{a}(\widehat{b}^\dagger + \widehat{b}) + \hbar\Delta|e\rangle\langle e| - \frac{\hbar\Omega}{2}\left[|e\rangle\langle g|e^{ik_x Y_{\mathrm{ZPF}}\left(\widehat{b}^\dagger + \widehat{b}\right)} + H.c.\right] . \quad \text{(S4)}$$

Derivation of Equation (1)

Interesting physics happens at the fluctuation about the averaged cavity photon number $\overline{n}_{cav}$ [1]. In this region, one can apply the replacement $\widehat{a} \rightarrow \sqrt{\overline{n}_{cav}} + \widehat{a}$ to Eq. (S4). The operator $\widehat{a}$ on the right hand side now denotes the new annihilation operator for photon number fluctuations around the average value $\overline{n}_{cav}$, namely, in the perturbation region. Also, in this region, the microlever's oscillation frequency, the mechanical damping rate and the optomechanical coupling constant are optomechanically modified as ω_m, $\gamma_0 + \delta\gamma_0$ and G, respectively [1],

$$\widehat{H} \;=\; \hbar\omega_m \widehat{b}^\dagger \widehat{b} - \hbar\Delta_c \widehat{a}^\dagger \widehat{a} - \hbar G\left(\widehat{a}^\dagger \widehat{b} + \widehat{a}\widehat{b}^\dagger\right) + \hbar\Delta|e\rangle\langle e| - \frac{\hbar\Omega}{2}\left[|e\rangle\langle g|e^{ik_x Y_{\mathrm{ZPF}}\left(\widehat{b}^\dagger + \widehat{b}\right)} + H.c.\right] . \quad \text{(S5)}$$

MASTER EQUATION

To calculate the Hamiltonian matrix elements, for $\eta\sqrt{n} < 1$, we can approximate

$$\begin{aligned} |e\rangle\langle g|e^{ik_x Y_{\mathrm{ZPF}}\left(\widehat{b}^\dagger + \widehat{b}\right)} &\simeq |e\rangle\langle g|\left[1 + ik_x Y_{\mathrm{ZPF}}\left(\widehat{b}^\dagger + \widehat{b}\right)\right], \\ &= |e\rangle\langle g|\left[1 + i\eta\left(\widehat{b}^\dagger + \widehat{b}\right)\right]. \end{aligned} \quad \text{(S6)}$$

Equation (S6) shows there are three paths for the system to reach the nuclear excited state $|e\rangle$ via absorbing an x-ray photon. The x-ray absorption can be accompanied by either deexciting a phonon, a zero phonon line or by exciting a phonon, as illustrated in Fig. S1. The corresponding Franck-Condon coefficients $F_n^m = \langle m|e^{i\eta\left(\widehat{b}^\dagger + \widehat{b}\right)}|n\rangle$ are

$$F_n^{m \geq n} \;=\; \frac{(i\eta)^{|m-n|}}{|m-n|!}\sqrt{\frac{m!}{n!}}, \quad \text{(S7)}$$

$$F_n^{m < n} \;=\; \frac{(i\eta)^{|m-n|}}{|m-n|!}\sqrt{\frac{n!}{m!}}. \quad \text{(S8)}$$

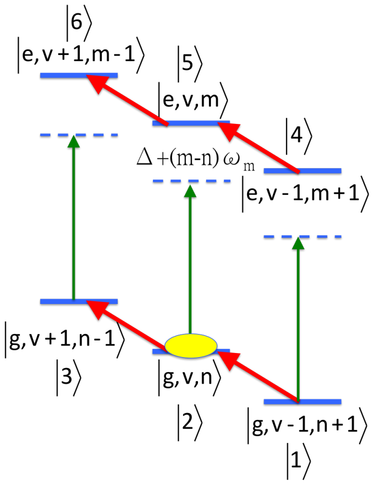

FIG. S1: Level scheme of the effective nuclear harmonic oscillator, adapted from Fig. 1b in the manuscript. Lower (upper) three states correspond to the ground (excited) state g (e) while v (n) denotes the number of cavity photons (number of phonons). Vertical green arrows depict the x-ray absorption by nuclei (with x-ray detuning Δ), and red diagonal arrows illustrate the beam splitter interaction between cavity photons and the microlever's mechanical motion. The full yellow ellipse indicates the initial state of the system. In derivations, for simplicity, we use state vectors labeled with numbers, i.e., $|1\rangle$, $|2\rangle$,$|3\rangle$,$|4\rangle$,$|5\rangle$, and $|6\rangle$.

Using the state notation introduced in Fig. S1, the explicit forms of each matrix in the master equation $\partial_t \widehat{\rho} = \frac{1}{i\hbar}\left[\widehat{H},\widehat{\rho}\right] + \widehat{\rho}_{dec}$ are given by

$$\widehat{H} = -\begin{bmatrix} (1-v)\Delta_c+(1+n)\omega_m & -G\sqrt{(1+n)v} & 0 & -\frac{1}{2}\Omega F_{n+1}^{m+1*} & 0 & 0 \\ -G\sqrt{(1+n)v} & -v\Delta_c+n\omega_m & -G\sqrt{n(1+v)} & 0 & -\frac{1}{2}\Omega F_n^{m*} & 0 \\ 0 & -G\sqrt{n(1+v)} & -(1+v)\Delta_c+(n-1)\omega_m & 0 & 0 & -\frac{1}{2}\Omega F_{n-1}^{m-1*} \\ -\frac{1}{2}\Omega F_{n+1}^{m+1} & 0 & 0 & -\Delta+(1-v)\Delta_c+(1+m)\omega_m & -G\sqrt{(1+m)v} & 0 \\ 0 & -\frac{1}{2}\Omega F_n^{m*} & 0 & -G\sqrt{(1+m)v} & -\Delta-v\Delta_c+m\omega_m & -G\sqrt{m(1+v)} \\ 0 & 0 & -\frac{1}{2}\Omega F_{n-1}^{m-1} & 0 & -G\sqrt{m(1+v)} & -\Delta-(1+v)\Delta_c+(m-1)\omega_m \end{bmatrix}, \quad \text{(S9)}$$

$$\widehat{\rho}_{dec} = \begin{bmatrix} (s-\frac{\Gamma}{2})\rho_{11}+\Gamma\rho_{44}+\kappa\rho_{22} & (s-\frac{\Gamma}{2})\rho_{12} & (s-\frac{\Gamma}{2})\rho_{13} & s\rho_{14} & s\rho_{15} & s\rho_{16} \\ (s-\frac{\Gamma}{2})\rho_{21} & (s-\frac{\Gamma}{2})\rho_{22}+\Gamma\rho_{55}+\kappa\rho_{33} & (s-\frac{\Gamma}{2})\rho_{23} & s\rho_{24} & s\rho_{25} & -s\rho_{26} \\ (s-\frac{\Gamma}{2})\rho_{31} & (s-\frac{\Gamma}{2})\rho_{32} & (s-\frac{\Gamma}{2})\rho_{33}+\Gamma\rho_{66} & s\rho_{34} & s\rho_{35} & s\rho_{36} \\ s\rho_{41} & s\rho_{42} & s\rho_{43} & (s+\frac{\Gamma}{2})\rho_{44}+\kappa\rho_{55} & (s+\frac{\Gamma}{2})\rho_{45} & (s+\frac{\Gamma}{2})\rho_{46} \\ s\rho_{51} & s\rho_{52} & s\rho_{53} & (s+\frac{\Gamma}{2})\rho_{54} & (s+\frac{\Gamma}{2})\rho_{55}+\kappa\rho_{66} & (s+\frac{\Gamma}{2})\rho_{56} \\ s\rho_{61} & s\rho_{62} & s\rho_{63} & (s+\frac{\Gamma}{2})\rho_{64} & (s+\frac{\Gamma}{2})\rho_{65} & (s+\frac{\Gamma}{2})\rho_{66} \end{bmatrix}, \quad \text{(S10)}$$

$$\widehat{\rho} = -\begin{bmatrix} \rho_{11} & \rho_{12} & \rho_{13} & \rho_{14} & \rho_{15} & \rho_{16} \\ \rho_{21} & \rho_{22} & \rho_{23} & \rho_{24} & \rho_{25} & \rho_{26} \\ \rho_{31} & \rho_{32} & \rho_{33} & \rho_{34} & \rho_{35} & \rho_{36} \\ \rho_{41} & \rho_{42} & \rho_{43} & \rho_{44} & \rho_{45} & \rho_{46} \\ \rho_{51} & \rho_{52} & \rho_{53} & \rho_{54} & \rho_{55} & \rho_{56} \\ \rho_{61} & \rho_{62} & \rho_{63} & \rho_{64} & \rho_{65} & \rho_{66} \end{bmatrix}. \tag{S11}$$

Typically, only low nuclear excitation is achieved in nuclear scattering with x-rays, such that the master equation in the perturbation region $\Gamma/2 + \kappa + \gamma_m > G \gg \Omega$ can be used. We assume all population $\rho_{ii}(t) = 0$ but $\rho_{22}(t) = 1$ due to the prepared initial condition. Also, we focus on the red-detuned regime, namely, cavity detuning $\Delta_c = -\omega_m$. In the perturbation regime, we neglect all the dynamics of populations and consider only coherences related to state $|2\rangle$:

$$\partial_t \rho_{21} = -(\gamma_m + \kappa)\rho_{21} - \frac{i}{2}\left[2G\sqrt{(1+n)v}\right], \tag{S12}$$

$$\partial_t \rho_{32} = -(\gamma_m + \kappa)\rho_{32} + \frac{i}{2}\left[2G\sqrt{(1+v)n}\right], \tag{S13}$$

$$\begin{aligned} \partial_t \rho_{42} = & -\left(\frac{\Gamma}{2} + \gamma_m + \kappa\right)\rho_{42} \\ & + \frac{i}{2}\left\{F_{n+1}^{m+1}\Omega\rho_{21}^{*} + 2G\sqrt{(1+m)v}\rho_{52} + 2\left[\Delta + (n-m)\omega_m\right]\rho_{42}\right\}, \end{aligned} \tag{S14}$$

$$\begin{aligned} \partial_t \rho_{52} = & -\left(\frac{\Gamma}{2} + \gamma_m + \kappa\right)\rho_{52} \\ & + \frac{i}{2}\left\{F_{n}^{m}\Omega + 2\left[\Delta + (n-m)\omega_m\right]\rho_{52} + 2G\left[\sqrt{(1+m)v}\rho_{42} + \sqrt{(1+v)m}\rho_{62}\right]\right\}, \end{aligned} \tag{S15}$$

$$\begin{aligned} \partial_t \rho_{62} = & -\left(\frac{\Gamma}{2} + \gamma_m + \kappa\right)\rho_{62} \\ & + \frac{i}{2}\left\{F_{n-1}^{m-1}\Omega\rho_{32} + 2G\sqrt{(1+v)m}\rho_{52} + 2\left[\Delta + (n-m)\omega_m\right]\rho_{62}\right\}. \end{aligned} \tag{S16}$$

Derivation of Equation (6)

The standard method to calculate the dispersion relation for x-rays is solving the steady state solution of the master equation, i.e., using $\partial_t \rho_{ij} = 0$ to get

$$0 = -(\gamma_m + \kappa)\rho_{21} - \frac{i}{2}\left[2G\sqrt{(1+n)v}\right], \tag{S17}$$

$$0 = -(\gamma_m + \kappa)\rho_{32} + \frac{i}{2}\left[2G\sqrt{(1+v)n}\right], \tag{S18}$$

$$\begin{aligned} 0 = & -\left(\frac{\Gamma}{2} + \gamma_m + \kappa\right)\rho_{42} \\ & + \frac{i}{2}\left\{F_{n+1}^{m+1}\Omega\rho_{21}^* + 2G\sqrt{(1+m)v}\rho_{52} + 2\left[\Delta + (n-m)\omega_m\right]\rho_{42}\right\}, \end{aligned} \tag{S19}$$

$$\begin{aligned} 0 = & -\left(\frac{\Gamma}{2} + \gamma_m + \kappa\right)\rho_{52} \\ & + \frac{i}{2}\left\{F_n^m\Omega + 2\left[\Delta + (n-m)\omega_m\right]\rho_{52} + 2G\left[\sqrt{(1+m)v}\rho_{42} + \sqrt{(1+v)m}\rho_{62}\right]\right\}, \end{aligned} \tag{S20}$$

$$\begin{aligned} 0 = & -\left(\frac{\Gamma}{2} + \gamma_m + \kappa\right)\rho_{62} \\ & + \frac{i}{2}\left\{F_{n-1}^{m-1}\Omega\rho_{32} + 2G\sqrt{(1+v)m}\rho_{52} + 2\left[\Delta + (n-m)\omega_m\right]\rho_{62}\right\}. \end{aligned} \tag{S21}$$

We thus obtain the most relevant coherence term ρ_{52}, since most of the population remains in state $|2\rangle$ during the period of interest. The real and imaginary parts of ρ_{52} are associated with dispersion and absorption, respectively [36],

$$\rho_{52}(\Delta) = \frac{\Omega\left\{F_n^m(2s-\Gamma)\left[is + \Delta - (m-n)\omega_m\right] - 2iG^2\left[F_{n+1}^{m+1}v\sqrt{(1+m)(1+n)} + F_{n-1}^{m-1}(1+v)\sqrt{mn}\right]\right\}}{2(2s-\Gamma)\left\{G^2(m+v+2mv) + \left[s - i(\Delta - (m-n)\omega_m)\right]^2\right\}}. \tag{S22}$$

When steady state arrives, $v = 0$ should be used and the coherence reads

$$\rho_{52}(\Delta) = \frac{\Omega\left\{F_n^m(2s-\Gamma)\left[is + \Delta - (m-n)\omega_m\right] - 2iG^2F_{n-1}^{m-1}\sqrt{mn}\right\}}{2(2s-\Gamma)\left\{G^2m + \left[s - i(\Delta - (m-n)\omega_m)\right]^2\right\}}. \tag{S23}$$

Here the asymmetric missing of F_{n+1}^{m+1} term is the consequence of the relation $\widehat{a}|0\rangle = 0$. Equation (S23) demonstrates that the x-ray spectra can be controlled by changing the optomechanical coupling constant via altering the averaged cavity photon number.

Eigensystem

With $s \gg \Omega$, the eigensystem for the setup is governed by the following simplified Hamiltonian

$$\widehat{H} = -\begin{bmatrix} (n+v)\omega_m & -G\sqrt{(1+n)v} & 0 & 0 & 0 & 0 \\ -G\sqrt{(1+n)v} & (n+v)\omega_m & -G\sqrt{n(1+v)} & 0 & 0 & 0 \\ 0 & -G\sqrt{n(1+v)} & (n+v)\omega_m & 0 & 0 & 0 \\ 0 & 0 & 0 & -\Delta+(m+v)\omega_m & -G\sqrt{(1+m)v} & 0 \\ 0 & 0 & 0 & -G\sqrt{(1+m)v} & -\Delta+(m+v)\omega_m & -G\sqrt{m(1+v)} \\ 0 & 0 & 0 & 0 & -G\sqrt{m(1+v)} & -\Delta+(m+v)\omega_m \end{bmatrix}. \quad \text{(S24)}$$

The corresponding eigenenergies and eigenstate vectors are presented in Table S2.

TABLE S2: Eigensystem of Hamiltonian Eq. (S11).

Eigenenergy	Eigenstate vector
$(n+v)\omega_m$	$\left(\sqrt{\frac{(1+v)n}{(1+n)v}},0,1,0,0,0\right)$
$-\Delta+(m+v)\omega_m$	$\left(0,0,0,\sqrt{\frac{(1+v)m}{(1+m)v}},0,1\right)$
$-G\sqrt{m+v+2mv}-\Delta+(m+v)\omega_m$	$\left(0,0,0,\sqrt{\frac{(1+m)v}{(1+v)m}},\sqrt{\frac{m+v+2mv}{(1+v)m}},1\right)$
$G\sqrt{m+v+2mv}-\Delta+(m+v)\omega_m$	$\left(0,0,0,\sqrt{\frac{(1+m)v}{(1+v)m}},-\sqrt{\frac{m+v+2mv}{(1+v)m}},1\right)$
$-G\sqrt{n+v+2nv}+(n+v)\omega_m$	$\left(\sqrt{\frac{(1+n)v}{(1+v)n}},\sqrt{\frac{n+v+2nv}{(1+v)n}},1,0,0,0\right)$
$G\sqrt{n+v+2nv}+(n+v)\omega_m$	$\left(\sqrt{\frac{(1+n)v}{(1+v)n}},-\sqrt{\frac{n+v+2nv}{(1+v)n}},1,0,0,0\right)$

OPTICAL THICKNESS ESTIMATE

The realistic microlever parameters considered in the manuscript were adopted from Ref. [38]. In particular, a microlever mass of $M = 0.14$ μg was considered in the numerical calculations. Here we determine the corresponding optical thickness values for the x-ray absorption. We consider the cases of ZnO, Ge and 10^{18} cm^{-3} Th nuclei doped in CaF_2 VUV-transparent crystals. The densities of these materials are 5.61 g/cm^3, 5.32 g/cm^3 and 3.18 g/cm^3, respectively. Depending on the exact sample geometry, the mass value $M = 0.14$ μg could correspond for example to a sample size of approx. 30x30x30 μm^3.

The optical thickness is given by $\xi = n\sigma l$, where n is the concentration, σ the resonant nuclear absorption cross section and l the thickness of a sample. The resonant nuclear cross section values are $\sigma_{\mathrm{ZnO}} = 5\times10^{-20}$ cm^2 [39], $\sigma_{\mathrm{Ge}} = 2\times10^{-20}$ cm^2 [39] and $\sigma_{\mathrm{Th}} = 10^{-10}$ cm^2 [40]. For ZnO and Ge we consider a number density of nuclei of 10^{22} cm^{-3}. The corresponding optical thickness ξ

is of order unity for ZnO and Ge and approx. 10^5 for 10^{18} cm^{-3} Th nuclei doped in CaF_2 crystals samples. By varying the ratio between sample concentration and sample thickness, larger optical thickness parameters can be achieved.
